# Nearly Half of Women Have Experienced Intimate Partner Violence During Pregnancy in Northwest Ethiopia, 2021; The Role of Social Support and Decision-Making Power

**DOI:** 10.3389/fpubh.2022.904792

**Published:** 2022-06-30

**Authors:** Azmeraw Ambachew Kebede, Mastewal Belayneh Aklil, Dereje Nibret Gessesse, Nuhamin Tesfa Tsega, Wubedle Zelalem Temesgan, Marta Yimam Abegaz, Tazeb Alemu Anteneh, Nebiyu Solomon Tibebu, Haymanot Nigatu Alemu, Tsion Tadesse Haile, Asmra Tesfahun Seyoum, Agumas Eskezia Tiguh, Ayenew Engida Yismaw, Goshu Nenko, Kindu Yinges Wondie, Birhan Tsegaw Taye, Muhabaw Shumye Mihret

**Affiliations:** ^1^Department of Clinical Midwifery, School of Midwifery, College of Medicine and Health Sciences, University of Gondar, Gondar, Ethiopia; ^2^Department of Women's and Family Health, School of Midwifery, College of Medicine and Health Sciences, University of Gondar, Gondar, Ethiopia; ^3^Department of Psychiatry, College of Medicine and Health Sciences, University of Gondar, Gondar, Ethiopia; ^4^School of Nursing and Midwifery, Asrat Woldeyes Health Science Campus, Debre Berhan University, Debre Birhan, Ethiopia

**Keywords:** Ethiopia, intimate partner, violence, social support, pregnancy

## Abstract

**Background:**

In developing countries, intimate partner violence is increasing alarmingly, though attention to this issue is rarely given. It has devastating effects on the general wellbeing of women, pregnancy outcomes, and the long-term health of children, and this needs to be addressed. Hence, this study was designed to assess intimate partner violence and associated factors in northwest Ethiopia.

**Methods:**

A community-based cross-sectional study was conducted from July 1^st^ to August 30^th^, 2021, among 858 postpartum women in Gondar city. A cluster sampling technique was employed to select the study participants. EPI DATA version 4.6 and SPSS 25 were used for data entry, cleaning and analysis, respectively. A bivariable and multivariable logistic regression model was fitted to identify factors associated with intimate partner violence. The level of significant association was declared using the adjusted odds ratio (AOR) with 95 % confidence interval (CI) and a *p*-value of ≤ 0.05.

**Results:**

In this study, 48.6% of women indicated having experienced intimate partner violence during pregnancy (95% CI: 45.3, 51.7). The odds of intimate partner violence during pregnancy were significantly higher among women who were not able to read and write (AOR = 4.96; 95% CI: 2.15, 11.41), were private workers (AOR = 1.78; 95% CI: 1.05, 3.02), and had low decision-making power (AOR = 1.43; 95% CI: 1.06, 1.95), a poor social support (AOR = 1.99; 95% CI: 1.32, 3.02), and unsupported pregnancy by family (AOR = 2.32; 95% CI: 1.26, 4.24). Whereas a family size of ≥ 5 (AOR = 0.73; 95% CI: 0.54, 0.98) appeared to be a protective factor for intimate partner violence.

**Conclusion:**

The magnitude of intimate partner violence was unacceptably high in the study area and connected to poor women's empowerment and social determinants of health. Thus, it is important to focus on interventions that improve women's access to social support and allow them to participate in all aspects of household decision-making through community-based structures and networks. It is also important to encourage women to improve their educational status and arrange risk-free employment opportunities.

## Introduction

Intimate partner violence (IPV) is a form of violence against a woman by her current or former spouse, leading to pain, psychological threat, and even death (1). It is one of the most common forms of violence against women (1, 2). Violence against women is increasing in Ethiopia, though little attention is paid to violence committed by intimate partners, especially against pregnant women (3). Pregnant women are more vulnerable psychologically and physically because of their pregnancy and IPV also to pose a risks to the unborn child (4, 5).

Existing evidence shows that three-quarters of women worldwide have experienced physical violence by their intimate partner; one-third of these women have been physically and/or sexually abused by their intimate partners (6). About 53.7% of women experienced either physical or sexual violence annually, and 70.9% of all women experience IPV during their lifetime (7). The magnitude of IPV ranged from 4–54% worldwide (8). Other studies conducted in Uganda and Gambia revealed IPV during pregnancy was 40.6 and 67%, respectively (9, 10). In Ethiopia, the pooled prevalence of IPV was 26.1% in 2018 (11).

Intimate partner violence (IPV) during pregnancy is associated with long-term effects on the physical and mental disorders of women and children (2, 3, 12). Some of the negative effects of IPV on women include pain, sexually transmitted infections (STI), traumatic fistula, inflammatory diseases of the pelvis (PID), and poor general health (13, 14). IPV is also has been associated with mental health disorders including anxiety, depression, suicidal ideation, and reduced health-seeking practices, poor motherhood, and addiction to different risky behaviors (i.e. drinking alcohol, cigarette smoking) (15–18). At its worst, IPV during pregnancy could result in unpleasant birth outcomes, such as fetal loss, low birth weight, premature birth, abortion, and infant death (19, 20).

Despite the endorsement of appropriate and effective legal policies to promote women's rights, combating violence against women is a major challenge in developing countries, including Ethiopia (21). Previous studies in Ethiopia identified that factors like lower women's educational level, lower decision-making power, poor social support, male dominance, alcohol consumption, and cigarette smoking by partners increase the occurrence of IPV during pregnancy (5, 7, 22, 23). Studies showed that economic dependence of wives on their husbands are at higher risk for IPV due to lack of respection, affordable housing options and poverty (24, 25).

Most of the previous studies evaluated IPV during pregnancy giving a special focus on physical violence. However, sexual and emotional violence are also considered to be determinants of the wellbeing of women and their children. This study stands in accordance with the recently adopted 2016 global agenda, which is aimed at ensuring women's empowerment and gender equality in every country (26). Moreover, research on violence against women, especially IPV during pregnancy, is limited in the study area. Therefore, understanding IPV and its determinant factors are imperative for a country like Ethiopia to design and combat violence against women during pregnancy, which in turn will improve maternal and child health. Hence, this study focused on assessing the prevalence of and factors associated with IPV during pregnancy in Gondar city, northwest Ethiopia. Based on our objectives we hypothesized the following point: Increasing women's education level, having high household decision making power and pregnancy supported by family have significant impact in reduction of IPV during pregnancy.

## Methods and Materials

### Study Design and Period

A community-based cross-sectional study was conducted in Gondar city from July 1^st^ to August 30^th^, 2021.

### Study Area

This study was conducted in Gondar city. The city is located in Central Gondar Zone, Amhara national regional state, northwest Ethiopia. It is located 750 km from Addis Ababa, the capital city of Ethiopia. There are 1 governmental referral hospital, 8 governmental health centers, 22 health posts, 1 private primary hospital, and 1 general hospital serving the city and other populations outside of the city. The recent estimated total population of the city is 432,191, of whom 224,508 females about 133, 477 (30.88%) of these females are in the reproductive age group (unpublished data by Amhara regional state, 2021).

### Study Population and Eligibility Criteria

All women who gave birth in the 6 months prior to data collection were the study population. All women residing for at least 6 months before the data collection period were included in the study.

### Sample Size Determination and Sampling Procedure

The single population formula was used to calculate the necessary sample size for the current study by considering the following assumptions: the proportion of IPV-44.5% (27), level of confidence-95%, and margin of error-5%. Therefore, the sample size (n) $=\frac{{\left(Z\frac{\alpha }{2}\right)}^{2^{*}p\left(1-p\right)}}{d^{2}}\;\frac{=\;{\left(1.96\right)}^{2^{*}}0.455\left(1-0.5\right)}{\left(0.05\right)2}$ = 380. Based on a design effect of 2 (since cluster sampling used) and a non-response rate of 10 %, a total sample size of 836 women needed.

In Gondar city, there are 22 kebeles (the smallest administrative unit in the government structure), from which 30% of the total kebeles (seven kebeles) were randomly selected by the lottery method. To reach out to the eligible women, a house-to-house visit was carried out in the selected kebeles (clusters). All women found to be eligible for the study were interviewed. Finally, due to the nature of cluster sampling, 858 women were included in the study.

### Variables of the Study

Intimate partner violence was the outcome variable, whereas women's age, women's occupation, monthly income, religion, women's educational status, marital status, family size, parity, antenatal care (ANC) visit, the number of ANC visits, whether the pregnancy was supported or not by family, pregnancy was planned or not, husband's educational status, husband's occupation, woman's household decision-making power, media exposure, and social support were the independent variables.

### Operational Definitions and Measurement

#### Intimate Partner Violence

Intimate partner is considered as a current spouse, co-habited partner, current boyfriends, or former partner or spouse. If the respondent said “Yes” to any one of the ranges of sexual, psychological, and physical or any combination of the three coercive acts regardless of the legal status of the relationship with current/former intimate partner, it was considered as intimate partner violence (28).

#### Household Decision-Making Power

The ability of women to act self-sufficiently about the household activities including their health, children's health, freedom of movement, and control over finance without needing permission from another person (29). Eight questions were prepared to assess the household decision-making power of women. The women's responses were coded 2 (if women decided independently), 1 (if women decided with their husbands), and 0 (if the decision was made by the husband or somebody else). The total score ranged from 0 to16. Thus, based on the summative score of variables designed to assess household decision-making power, women who were answered above the mean value were considered as having higher decision-making power (29, 30).

#### Social Support

The Oslo-3 Social Support Scale (OSSS-3) was used to measure social support. The scale consists of 3 items addressing the number of close intimates to the woman, perceived level of concern from others, and perceived easiness of getting support from neighbors. Accordingly, the level of social support was categorized as “poor” with a score of 3–8; “moderate” with a score of 9–11; and “strong” with a score of 12–14 (31).

#### Mass Media Exposure

Study participants were asked how often they watched television, read a newspaper, or listened to the radio. Respondents who responded at least once a week are considered to be regularly exposed to that form of media (21).

### Data Collection Procedures

After reviewing related literature and WHO multi country study on women's health and domestic violence (5, 8, 27, 28) the data collection instrument was developed to elicit required information on IPV. The English version of the questionnaire was prepared first and translated to the local language (Amharic) and back English to ensure its consistency. Data were collected using a structured and interviewer-administered questionnaire through face-to-face interviews. The questionnaire contains socio-demographic characteristics, obstetric and maternal health services-related characteristics, decision-making power, social support, and intimate partner violence-related questions. A total of 14 BSc and 4 MSc in Midwifery holders were involved in the data collection and supervision process, respectively. The one-day training was provided about the interview technique and information-handling techniques. The pretest was done on 5% (*n* = 42) of the calculated sample size outside of the study site. The language clarity and validity of the tool were checked and necessary revisions were made after the pretest. The supervisors checked for questionnaire completeness daily.

### Data Management and Analysis

Data were checked, coded, and entered into EPI DATA version 4.6 and further cleaning and analysis were done using SPSS version 25. Binary logistic regression analysis was performed to identify candidate predictors. Predictors with a *p*-value of < 0.25 were included in the multivariable logistic regression analysis to address the possible effect of confounders. In the last model (multivariable logistic regression analysis,) a *p*-value of ≤ 0.05 with 95% CI for the AOR was used to decide the level of association. The variance inflation factor (VIF) was used to check the multicollinearity assumption and was acceptable with a value of < 10. The Hosmer Lemeshow goodness-of-fit was performed to check the model fit.

## Results

### Sociodemographic Characteristics of the Study Participants

A total of 858 postpartum women were included in the analysis, a response rate of 98.4%. The mean age of the study participants was 29.53 years (±4.79SD) and about 43.2% of them were classified in the age group of 24–29 years old. More than two-fifths (43.2%) of the women had earned a diploma and above education and about 44.4% of women were housewives by occupation (Table 1).

**Table 1:** Socio-demographic characteristics of the study participants in Gondar city, northwest Ethiopia, 2021 (*n* = 858).

**Variables**	**Frequency**	**Percentage (%)**
**Age of women**		
18–23	74	8.6
24–29	371	43.2
30–35	319	37.2
≥36	94	11.0
**Women's educational level**		
Can't read and write	50	5.9
Can read and write	55	6.4
Primary education	140	16.3
Secondary education	242	28.2
Diploma and above education level	371	43.2
**Women's occupation**		
House wife	381	44.4
Merchant	105	12.2
Government employed	239	27.9
Private employed	99	11.5
Student	34	4.0
**Religion**		
Orthodox	706	82.3
Muslim	107	12.5
Others^*^	45	5.2
**Marital status**		
Married	779	90.8
Unmarried	79	9.2
**Husband educational level** (*n =* 779)		
No formal education	49	6.3
Primary education	55	7.0
Secondary education	165	21.2
Diploma and above education level	510	65.5
**Husband occupation** (*n =* 779)		
Daily laborer	88	11.3
Merchant	172	22.0
Private employed	164	21.1
Government employed	355	45.6
**Household average monthly income**		
<2500 ETB	116	13.5
≥2500 ETB	742	86.5
**Family size**		
1–4	488	56.9
≥5	370	43.1
**Mass media exposure**		
Yes	706	82.3
No	152	17.7
**Have medical problems**		
Yes	86	10.0
No	772	90.0

* *Protestant and catholic. ETB, Ethiopian Birr*.

### Reproductive and Maternity Health Characteristics

Most (97.3%) of the study participants had ANC follow-up during their last pregnancy. More than four-fifths (86.8%) of the study participants had planned pregnancies. About 28.8% of the women had poor social support (Table 2).

**Table 2:** Reproductive and maternity health service characteristics of the study participants in Gondar city, (*n* = 858).

**Variables**	**Frequency**	**Percent (%)**
**ANC follow up**		
Yes	835	97.3
No	23	2.7
**Number of ANC visit (*****n =*** **835)**		
1–3	320	38.3
≥4	515	61.7
**Parity**		
1–2	602	70.2
≥3	256	29.8
**Last pregnancy planned**		
Yes	745	86.8
No	113	13.2
**Last pregnancy supported by family**		
Yes	794	92.5
No	64	7.5
**Household decision-making power**		
Low	343	40
High	515	60
**Social support**		
Poor support	247	28.8
Moderate support	392	45.7
Strong support	219	25.5

*ANC, Antenatal care*.

### Intimate Partner Violence and Associated Factors

**Figure 1:**
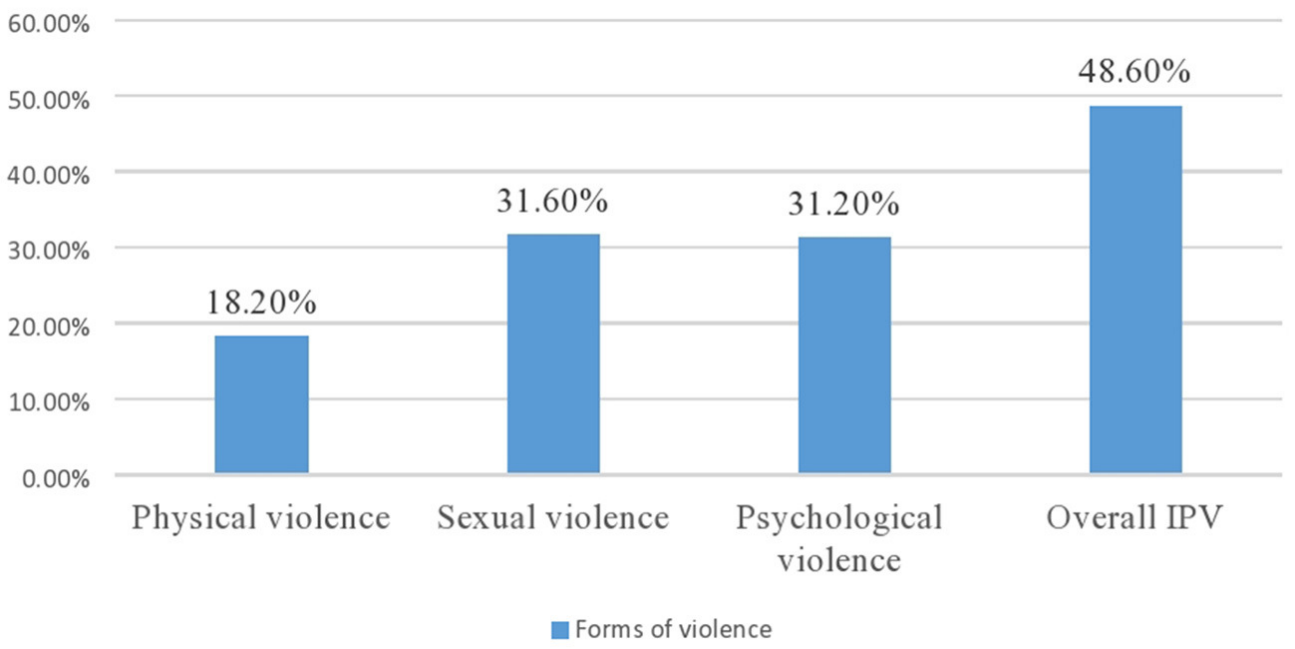
Forms of intimate partner violence during pregnancy in Gondar city, northwest Ethiopia, 2021.

In the present study, about 48.6% of the women indicated having experienced IPV during pregnancy (95% CI: 45.3, 51.7) (Figure 1). On bivariable analysis, age of women, women's education, women's occupation, husband education, husband occupation, household decision making power, social support, average monthly income, had ANC follow up, number of family and pregnancy supported family were significantly associated with intimate partner violence during pregnancy at *p*-value < 0.25. However, woman's education, woman's occupation, woman's decision-making power, woman's social support, family size, and pregnancy supported by families were the factors significantly associated with IPV in multivariable regression.

Accordingly, women who were unable to read and write were 4.96 times more likely to report experiencing IPV than women who held a diploma and above education (AOR = 4.96; 95% CI: 2.15, 11.41). Likewise, privately -employed women were 1.78 times more likely to report experiencing IPV compared with government employed women (AOR = 1.78; 95% CI: 1.05, 3.02).

This study revealed that women who had a family size of ≥ 5 were 27% less likely to report experiencing IPV compared with those women who had a family size of 1–4 (AOR = 0.73; 95% CI: 0.54, 0.98). Similarly, women who had low decision-making power had 1.43 times higher odds of experiencing IPV compared with those women having higher decision-making power **(**AOR = 1.43; 95% CI: 1.06, 1.95). This study also found that the odds of experiencing IPV among women who had poor social support were two times higher (AOR = 1.99; 95% CI: 1.32, 3.02) than women who had strong social support. Moreover, women whose pregnancy was unsupported by their husband or family were 2.32 times more likely to report experiencing IPV during pregnancy as compared to women whose pregnancy was supported by their husband or family (AOR = 2.32; 95% CI: 1.26, 4.24) (Table 3).

**Table 3:** Bivariable and multivariable logistic regression analysis of factors associated with intimate partner violence among pregnant women in Gondar city, northwest Ethiopia, 2021.

**Variables**	**Intimate partner**	**violence**	**COR (95% CI)**	**AOR (95% CI)**
	**Yes**	**No**		
**Age of women**				
18–23	38	36	0.89 (0.48, 1.63)	0.54 (0.26, 1.12)
24–29	188	183	0.86 (0.55, 1.36)	0.72 (0.42, 1.24)
30–35	140	179	0.65 (0.41, 1.04)	0.70 (0.42, 1.16)
≥ 36	51	43	1	1
**Women's education status**				
Can't read and write	41	9	7.34 (3.46, 15.57)	4.96 (2.15, 11.41)^*^
Able to read and write	31	24	2.08 (1.17, 3.69)	1.61(0.83, 3.09)
Primary education	72	68	1.70 (1.15, 2.52)	1.26 (0.77, 2.06)
Secondary education	131	111	1.90 (1.37, 2.64)	1.46 (0.96, 1.23)
Diploma and above education level	142	229	1	1
**Women's occupation**				
House wife	204	177	2.12 (1.52, 2.96)	1.23 (0.77, 1.95)
Merchant	55	50	2.03 (1.27, 3.23)	1.65 (0.95, 2.89)
Student	17	17	1.84 (0.89, 3.80)	0.61 (0.26, 1.43)
Private employed	57	42	2.50 (1.55, 4.04)	1.78 (1.05, 3.02)^*^
Government employed	84	155	1	1
**Husband education**				
No formal education	31	18	2.08 (1.13, 3.81)	0.79 (0.34, 1.81)
Primary education	31	24	1.56 (0.89, 2.73)	0.67 (0.33, 1.34)
Secondary education	85	80	1.28 (0.90, 1.82)	0.80 (0.51, 1.27)
Diploma and above education level	231	279	1	1
**Husband/partner occupation**				
Daily laborer	52	36	1.92 (1.20, 3.09)	1.06 (0.61, 1.82)
Merchant	91	81	1.50 (1.04, 2.16)	1.39 (0.92, 1.09)
Private employed	83	81	1.36 (0.94, 1.98)	1.01(0.67, 1.51)
Government employed	152	203	1	1
**Household decision-making power**				
Low	195	148	1.73 (1.32, 2.29)	1.43 (1.06, 1.93)^*^
High	222	293	1	1
**Social support**				
Poor	150	97	2.58 (1.77, 3.75)	1.99 (1.32, 3.02)^**^
Moderate	185	207	1.49 (1.06, 2.09)	1.38 (0.96, 1.97)
Strong	82	137	1	1
**Average monthly income**				
<2500 ETB	67	49	1.53 (1.03, 2.27)	0.75 (0.46, 1.22)
≥ 2500 ETB	350	392	1	1
**Have ANC follow up**				
Yes	402	433	1	1
No	15	8	2.02 (0.84,4.81)	1.45 (0.55, 3.77)
**Number of family**				
1–4	257	231	1	1
≥ 5	160	210	0.68 (0.52, 0.89)	0.73 (0.54, 0.98)^*^
**Pregnancy supported by family**				
Yes	371	423	1	1
No	46	18	2.91(1.66, 5.11)	2.32 (1.26, 4.24)^*^

** *p ≤ 0.001*,

* *p ≤ 0.05. ANC, Antenatal care; AOR, adjusted odds ratio; COR, crude odds ratio; CI, confidence interval; ETB, Ethiopian Birr; 1, Reference category*.

## Discussion

Intimate partner violence is a major public health concern that needs urgent policy action and multi-sectorial collaboration. Despite an endeavor made to overcome violence against women globally, it is still very much common in developing countries, including Ethiopia. One of the global goals set to be achieved by 2030 is empowering women and ensuring gender equality everywhere (26). Hence, this study focused on assessing the magnitude of IPV during pregnancy and associated factors in Gondar city, northwest Ethiopia.

In this study, about 48.6% of women reported having experienced IPV during pregnancy. This finding is higher than previous studies conducted in Uganda-40.6% (9), Nigeria-33% (32), and Vietnam-35.2% (33). The results of the current study show IPV in Gondar city to be higher than in studies conducted elsewhere in Ethiopia, including Debre markos town-41.1% (28), Shire Enda Selassie town-20.6% (5), and Western Ethiopia-44.5% (27). The possible explanation for the variation might be differences in the study population, time of data collection, and the instrument we used to measure the outcome variable. The study population in the aforementioned studies included women who came for antenatal care (ANC). According to scholars, IPV impacts the utilization of maternal health services, by women who have suffered from IPV. These women might be reluctant to visit the ANC clinic (34). Moreover, the above-mentioned studies included women all trimesters of the pregnancy and some women may experience violence at any time during pregnancy. In contrast, the current study included women after delivery which could be responsible for the higher magnitude of IPV. In addition, the current data were collected during the coronavirus disease 2019 (COVID-19) pandemic. According to empirical evidence, IPV has increased due to the lockdown (35).Another explanation for the high prevalence IPV-rate found in the current study is that IPV was measured using three forms of violence; sexual, physical, and psychological, instead off only physical IPV generally used in other studies (e.g., Shire Enda Selassie town).

There are also studies conducted in other African countries mentioning a higher IPV prevalence rate than found in our study. For instance, in Kenya-66.9% (36) and Gambia-67% (10). This variation could be due to the fact that women's health is getting global attention and violence against women is nowadays considered to be a violation of human rights. The other explanation for the disparity might be related to women's employment status; the majority (75%) of women from Kenya were unemployed suggesting that they could be financially dependent on their sexual partners/husbands. Evidence supports that being unable to secure financial needs and being jobless could increase the likelihood of one form of violence (37, 38). In this regard, concerned stakeholders and human rights commissions need to prepare an assessment instrument that will reach out to all women in the maternal continuum of care and the devastating nature of IPV should be advocated within the community.

This study points out that the odds of falling victim of IPV was 1.43 times higher among women with less decision-making power than among women with more decision-making power. This finding contradicts a study conducted in Sub-Saharan Africa, which reported that women who could make decisions were more likely to experience IPV (37). African culture, particularly Ethiopian culture, is gender stratified, in favor of men, and women are subordinated to men. However, in the present study, less autonomous women were more at risk of falling victims of IPV. This could be since most men in developing countries consider themselves as the main source of household income and they perceive they have every right to violet the women. Besides, less autonomous women are expected to be financially dependent on their partners. According to evidence, being economically dependent increases the odds of experiencing IPV. This finding is supported by a previous study, in which decisions made by the women alone or jointly with their partners had a positive impact on decreasing IPV (39).

The results of the present study also affirmed that social support during pregnancy was significantly associated with IPV. Accordingly, the odds of experiencing IPV were nearly 2 times higher among women who had poor social support as compared to those women who had strong social support. This finding is in agreement with a previous study done in Vietnam (33). According to scholars, social support helps women maintain positive self-esteem and a negative attitude toward IPV, thereby preparing them to solve the conflict of opinions meticulously with their sexual partners (40).

In the present study the odds of experiencing IPV among women who had unsupported pregnancies by families were 2.32 times higher compared with women who were pregnant with support of their families. This finding is consistent with a previous study conducted in Ethiopia (41). Unplanned and therefore unsupported pregnancies could push men to put the responsibility of pregnancy on women and to use violence. Evidence supported that unplanned and undesired pregnancies by partners are common reasons for committing violence against pregnant women (42). It should be noted, that no matter how the pregnancy happened, pregnant women must get respect and appropriate care to have an optimal outcome of the pregnancy and to ensure the wellbeing of the women. To reach this goal, adequate counseling should be offered to male partners on every occasion, with special focus on pregnancies that are unplanned and unsupported.

The present study revealed that the likelihood of experiencing IPV was nearly 5 times higher among women who were unable to read and write than those women attending diploma and above education. This finding is supported by a previous study conducted in Ethiopia where women who had no formal education were victims of IPV (5). This could be due to the reality that less-educated women might have limited access to any information related to their rights and women's empowerment. In addition, less educated women might be more adherent to the local cultures and may accept IPV as normal if it is committed by a formal sexual partner or husband (43).

The odds of experiencing IPV among women having a family size of ≥ 5 were 27% less likely compared with their counterparts. This could be due to the fact that women with a higher family size are expected to be older and therefore get more respect from their husbands and make shared decisions in every aspect of the household. Women with bigger families are expected to have strong social support and social networks, thereby maintaining smooth relationships with their sexual partners/husbands (44).

In the present study, women who were working in the private sector were 1.78 times more likely to experience IPV than those women who were government employees. In the private-sector employees are stimulated to focus on what makes the organization profitable. To do this, the staff can interact with a large number of people in order to attract customers; however, their sexual partners may interpret this in a negative way and this could turn into abusive behaviors.

### Limitations of the Study

The present study has limitations. Social desirability and recall biases might have been present. However, the study participants had been informed that their participation was crucial for the study, that the information was anonymous, and kept confidential for the study purpose only. Despite these limitations, the findings from study provide important insights for policy action related to violence against women.

## Conclusion

The magnitude of IPV during pregnancy was high. Being unable to read and write, being a private worker, having low-decision-making power, having poor social support, unsupported pregnancy, and having a bigger family were the factors significantly associated with IPV. Concerned stakeholders need to pay special attention to ensuring women's empowerment and gender equality by establishing social support platforms, engaging women in decision-making matters, and capacitating women financially by arranging risk-free employment.

## Data Availability Statement

The raw data supporting the conclusions of this article will be made available by the authors, without undue reservation.

## Ethics Statement

Ethical approval for the study was obtained from the Institutional Review Board (IRB) of the University of Gondar (Reference No: V/P/RCS/05/2710/2021). The patients/participants provided their written informed consent to participate in this study.

## Author Contributions

AK conceived and designed the experiments. DG, NTs, MAk, WT, MAb, TA, NTi, HA, TH, AS, AT, AY, MM, GN, KW, and BT investigation, analyzed and interpreted the data, and wrote the article. All authors contributed to the article and approved the submitted version.

## Conflict of Interest

The authors declare that the research was conducted in the absence of any commercial or financial relationships that could be construed as a potential conflict of interest.

## Publisher's Note

All claims expressed in this article are solely those of the authors and do not necessarily represent those of their affiliated organizations, or those of the publisher, the editors and the reviewers. Any product that may be evaluated in this article, or claim that may be made by its manufacturer, is not guaranteed or endorsed by the publisher.
